# Molecular assembly of amino acid interlinked, topologically symmetric, π-complementary donor–acceptor–donor triads

**DOI:** 10.3762/bjoc.9.178

**Published:** 2013-08-01

**Authors:** M B Avinash, K V Sandeepa, T Govindaraju

**Affiliations:** 1 Bioorganic Chemistry Laboratory, New Chemistry Unit, Jawaharlal Nehru Centre for Advanced Scientific Research, Jakkur, Bangalore-560064, India. Fax: +91 80 2208 2627; Tel: +91 80 2208 2969

**Keywords:** amino acids, charge transfer, designer functional molecules, molecular assembly, molecular recognition

## Abstract

Amino acid interlinked pyrene and naphthalenediimide (NDI) based novel donor–acceptor–donor (D-A-D) triads are designed to exploit their topological symmetry and complementary π-character for facile charge-transfer complexation. Consequently, free-floating high-aspect-ratio supercoiled nanofibres and hierarchical helical bundles of triads are realized by modulating the chemical functionality of interlinking amino acids.

## Findings

**Figure 1:**
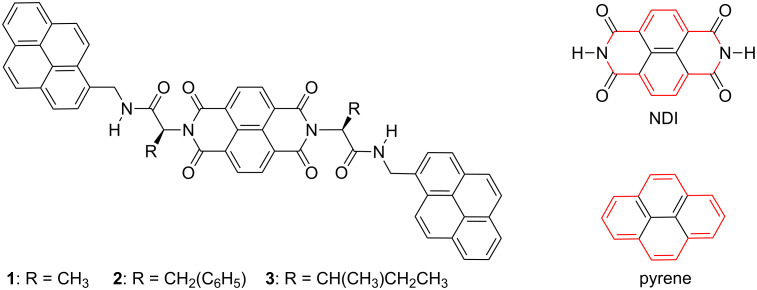
Molecular structures of donor–acceptor–donor traids. The red colouring highlights the topological symmetry of NDI and pyrene.

Geometric shapes, size and patterns have attracted the inquisitive human mind since time immemorial [1–2]. The significance of geometric complementarity and chemical functionality can be exemplified by the well-known “lock and key” mechanism of enzymes as well as the prototypal molecular recognition manifested in biochemical processes [1–4]. It is this elementary yet elegant design strategy involving topological symmetry of molecular structure that is explored in this current communication. Herein, we have employed NDI as the functional molecule due to its potential applications in field-effect transistors, photovoltaics and flexible displays [5–6]. Since the performance of electro-active molecular materials [7–10] is essentially dependant on their molecular assembly [11–16], it is extremely important to identify suitable donor–acceptor sequences for efficient energy or charge-transfer processes. It is with this conception that we have designed pyrene based D-A-D triads due to the topological symmetry of pyrene with that of NDI (Figure 1).

A variety of D-A systems [17–33] comprising pyrene and/or NDI have been reported in the literature, viz., supramolecular n/p heterojunctions [17], adhesive π-clamping [18], catenane [19], rotaxane [20], self-healable supramolecular polymer [21], aromatic stacking within a coordination cage [22], superamphiphile [23] and thermochromic [24] materials. Notably, Wilson et al. have reported preferential π-stacking of pyrene and NDI amongst a pool of π-electron D-A combinations, when tethered to a polymer or oligomer [2]. This preferential aromatic interaction was proposed to be a consequence of frontier orbital congruence for the HOMO of pyrene with the LUMO of NDI. Alternatively, NDI is one of the highly π-acidic molecules with an estimated molecular quadrupole moment (*Q_zz_*) of +18.6 B (Buckinghams), which is in the range of the explosive TNT [34]. This π-acidic behaviour of NDI is an interesting property, because most of the aromatic compounds are π-basic, viz., benzene has *Q_zz_* = −8.5 B, whereas pyrene has *Q_zz_* = −13.8 B. Thus, pyrene and NDI represent a unique D-A system due to topological symmetry in their molecular structure and complementary π-character. Since NDI is one of the most promising organic n-type semiconductors, we anticipated that alternative stacking with a special donor such as pyrene would lead to facile charge-transfer interactions. Moreover, 1D organization of D-A stacks could bring about significant improvement in the conductivity through directional charge transfer. Herein, we have chosen three D-A-D triads (**1**–**3**) of pyrene and NDI interlinked by different amino acids, namely alanine, phenylalanine and isoleucine (Figure 1). The choice of amino acids as linkers is due to their compactness, variable side chain functionality and sequence-specific self-assembling properties and the desire to exploit their inherent chiral information for helical assembly of electro-active molecular materials [35–48].

**Figure 2:**
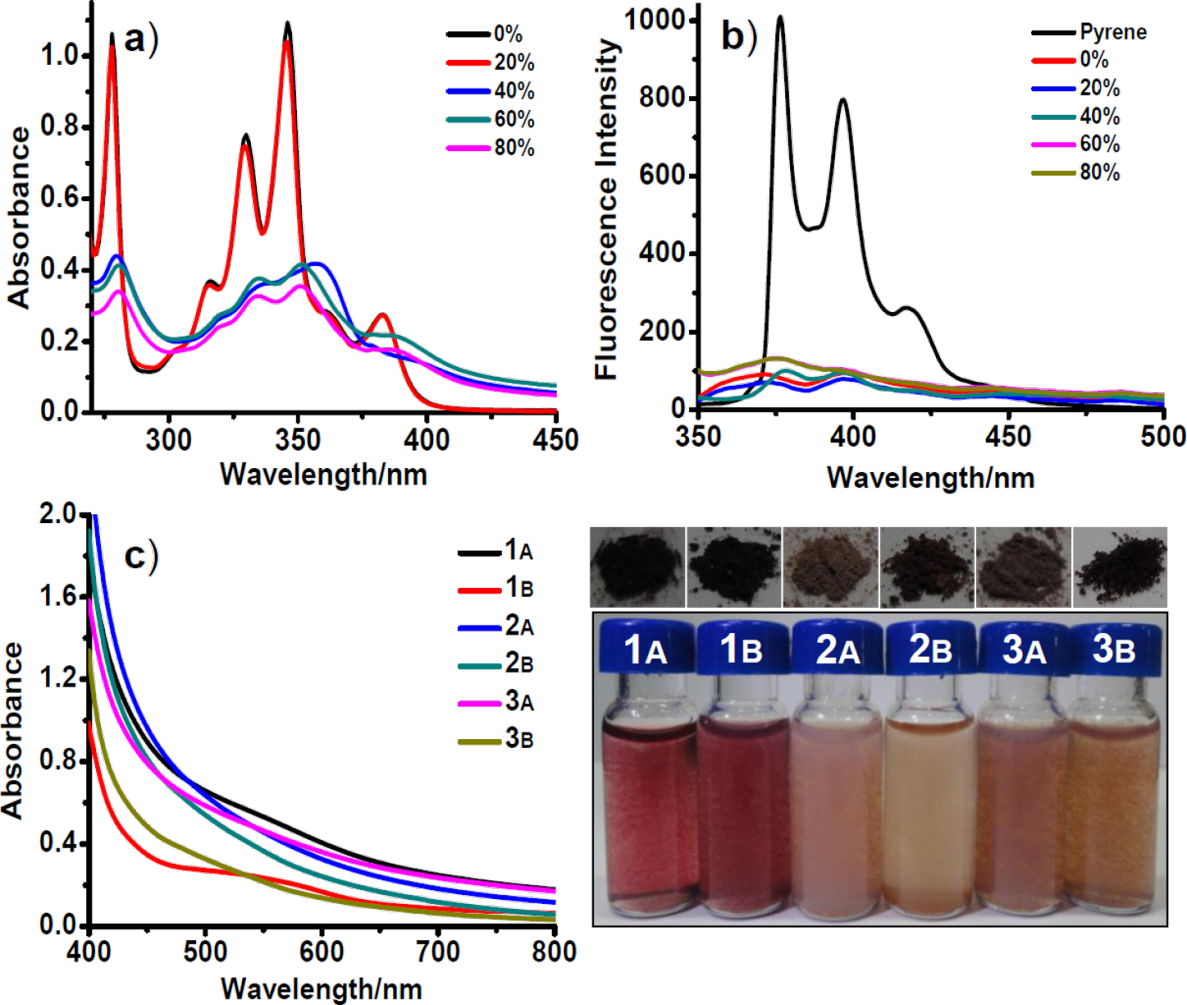
(a) UV–vis and (b) fluorescence spectra of **1** (200 μM) in aqueous DMSO for various percentages of water in DMSO. Excitation at 345 nm. For comparison fluorescence emission spectra of pyrenemethylamine hydrochloride is shown (black trace, pyrene) in (b). (c) UV–vis spectra of 1 mM solutions of **1**, **2** and **3** in 40% aqueous DMSO/NMP show the charge-transfer bands. The photograph shows the solid-state and free-floating aggregates of **1**, **2** and **3** with their characteristic colours due to charge-transfer complexation. A: 40% aqueous DMSO. B: 40% aqueous NMP.

A detailed procedure for the synthesis of the triads **1**, **2** and **3** is given in Supporting Information File 1. Briefly, 1,4,5,8-naphthalenetetracarboxylic acid dianhydride was condensed with amino acids (alanine/phenylalanine/isoleucine) to give the respective symmetrically substituted NDIs, which were then subjected to amide coupling with pyrenemethylamine to obtain the triads (**1**–**3**) in good yield. UV–vis absorption spectra of **1** in dimethyl sulfoxide (DMSO) exhibit absorption bands in the region of 270–400 nm due to characteristic π–π* transitions of pyrene and NDI (Figure 2a). With the successive addition of water to the solution of **1** in DMSO, a decrease in absorption intensity as well as broadening of the bands was observed. Interestingly, at 40% aqueous DMSO, a red shift of ~13 nm was observed for the absorption band at 346 nm (λ_max_). However, at 80% aqueous DMSO a blue shift of ~8 nm was observed with a net red shift of ~5 nm with respect to the absorption maxima in DMSO. This could be ascribed to the formation of offset type stacking of the triad due to solvatophobic interactions at 40% aqueous DMSO, whereas the enhanced hydrophobic forces at 80% aqueous DMSO reduce the offset by forming a stronger charge-transfer complex. Similar variations in the absorption spectra were observed for **2** in aqueous DMSO, while **3** exhibits only an incremental red shift of absorption maxima without any indication of the existence of two different (described above) kinds of aggregates as observed for **1** (see Supporting Information File 1).

On the contrary, in aqueous *N*-methyl-2-pyrrolidone (NMP) solvent system, **1** undergoes a decrease in absorption intensity until 60% aqueous NMP followed by a net red shift of ~5 nm at 80% aqueous NMP (see Supporting Information File 1). However, **2** and **3** in aqueous NMP exhibited only an incremental red shift of absorption maxima similar to that observed in the case of **3**, in aqueous DMSO (see Supporting Information File 1). In addition, the fluorescence emission for **1**, **2** and **3** was found to be quenched in DMSO and NMP as well as in their aqueous medium (Figure 2b). Interestingly, in the case of **2**, (both in aqueous DMSO as well as in aqueous NMP) a new weak emission band at ~460 nm was observed, which was attributed to the excimer of pyrene (see Supporting Information File 1). In addition, UV–vis spectra clearly show a new broad absorption band in the range of 450 nm to 700 nm confirming the charge-transfer complexation of pyrene and NDI (Figure 2c) [2]. Consequently, aqueous solutions of **1**, **2** and **3** exhibited the effect of interlinking (amino acid) chemical functionality by their characteristic dark red, light pink and brownish red colouration, respectively (Figure 2).

**Figure 3:**
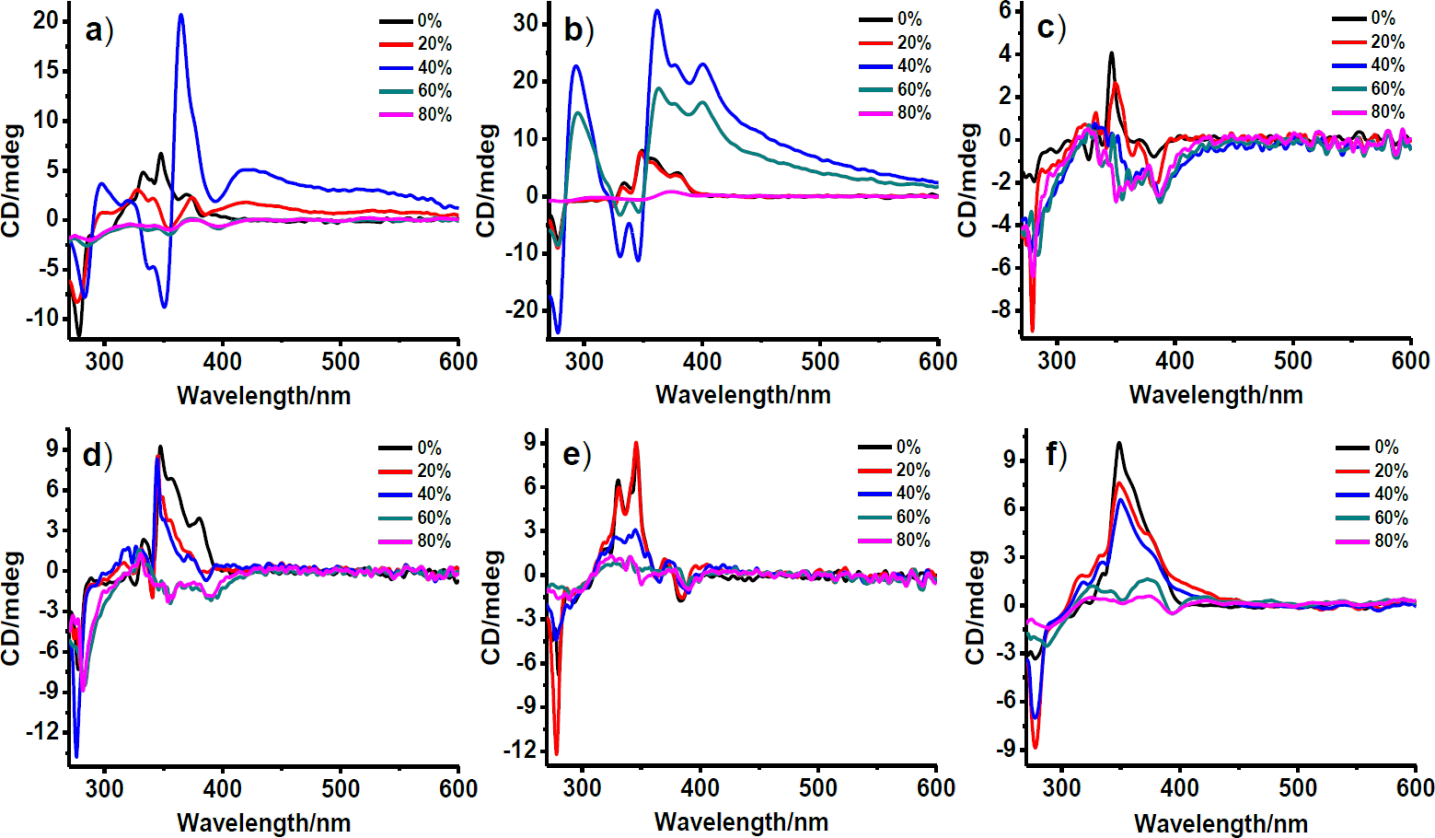
CD spectra of (a) and (b) **1**, (c) and (d) **2**, and (e) and (f) **3** for various percentages of water in aqueous DMSO (a, c and e) and aqueous NMP (b, d and f). Concentration of **1**, **2** and **3** used was 400 μM.

In order to gain further insights into the nature of molecular interactions, **1**, **2** and **3** were subjected to circular dichroism (CD) spectroscopic studies. The CD spectra of **1** in DMSO show positive excitonic cotton effects, indicating a P-type helical assembly (Figure 3a). However, with increase in the percentage of water in DMSO a bisignated cotton effect was observed in the range of 300–400 nm, besides the appearance of two new bands at ~420 nm and ~550 nm. The band at ~420 nm could be attributed to the aggregation band of NDI, while the band at ~550 nm is assigned to the charge-transfer complexation of NDI and pyrene. With further increase in the percentage of added water in DMSO, the amplitude of the CD signal approaches zero, suggesting a probable CD silencing [37]. Similar changes in the CD spectra were observed for **1** in aqueous NMP (Figure 3b). In contrast, **2** and **3** show significantly distinct excitonic cotton effects (Figure 3c–f). In DMSO, **2** (Figure 3c) and **3** (Figure 3e) exhibit a weak CD signal above 350 nm suggesting minimal excitonic coupling of NDI chromophores. However, CD studies for **2** (Figure 3d) and **3** (Figure 3f) in NMP show positive excitonic cotton effects indicating P-type helicity. In aqueous DMSO as well as in aqueous NMP both **2** and **3** tend to approach towards CD silencing. The relatively bulkier side chain functionality of the interlinking amino acids in **2** and **3** is believed to inhibit the effective excitonic coupling of the chromophores in lower percentages (≤ 60%) of the aqueous solution. However, at higher percentages of the aqueous solution the enhanced hydrophobic forces render transition of the angles between transition dipole moments of the stacked chromophores towards zero, resulting in CD silencing. Moreover, the relative differences between the CD features of **2** and **3** in aqueous DMSO and aqueous NMP could be ascribed to solvatophobic interactions.

**Figure 4:**
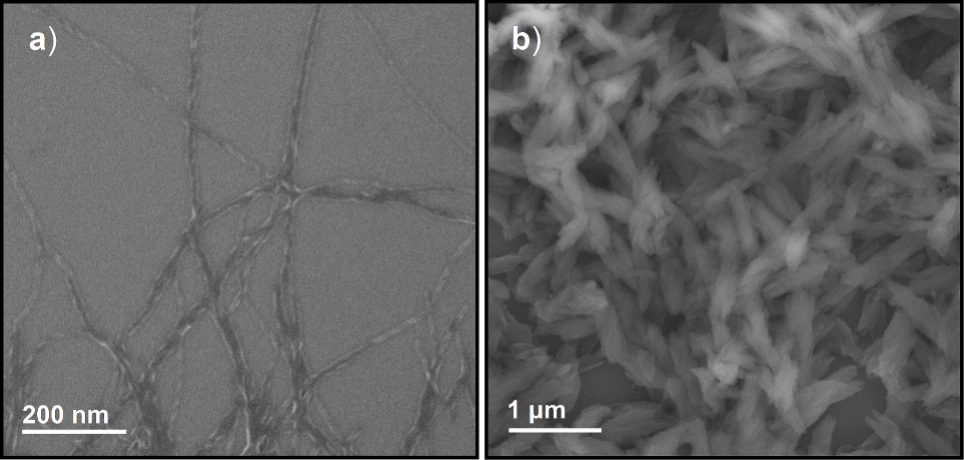
FESEM image of (a) **1** obtained from 40% aqueous NMP, (b) **2** obtained from 80% aqueous NMP.

2D NOESY studies of **1**, **2** and **3** in DMSO revealed the presence of weak aromatic interactions between pyrene and NDI (see Supporting Information File 1). Moreover the amide NH proton of **1**, **2** and **3** showed spatial interactions with the proton of the α-carbon and the side chain functionality of the interlinking amino acid, which suggests a *trans* configuration for the amide group. Powder X-ray diffraction (PXRD) studies for aggregates of **1**, **2** and **3** showed a broad peak corresponding to d-spacing of ~3.4 Å, indicating a strong aromatic stacking (see Supporting Information File 1). Remarkably **1**, **2** and **3** form free floating aggregates within a couple of hours in aqueous DMSO and aqueous NMP (Figure 2). These free floating aggregates were subjected to morphological studies using field emission scanning electron microscopy (FESEM). FESEM images of **1** in 40% aqueous NMP showed the presence of supercoiled nanofibres of ~10 nm diameter and a helical pitch of ~30 nm (Figure 4a). At 80% aqueous NMP, a dense network of nanofibres of **1** was observed (see Supporting Information File 1). Interestingly, at 80% aqueous NMP, **2** was found to form hierarchical helical bundles of ~1 μm length and ~200 nm diameter (Figure 4b). However, **3** showed only the presence of spherical or random aggregates both in aqueous DMSO as well as in aqueous NMP (see Supporting Information File 1).

**Figure 5:**
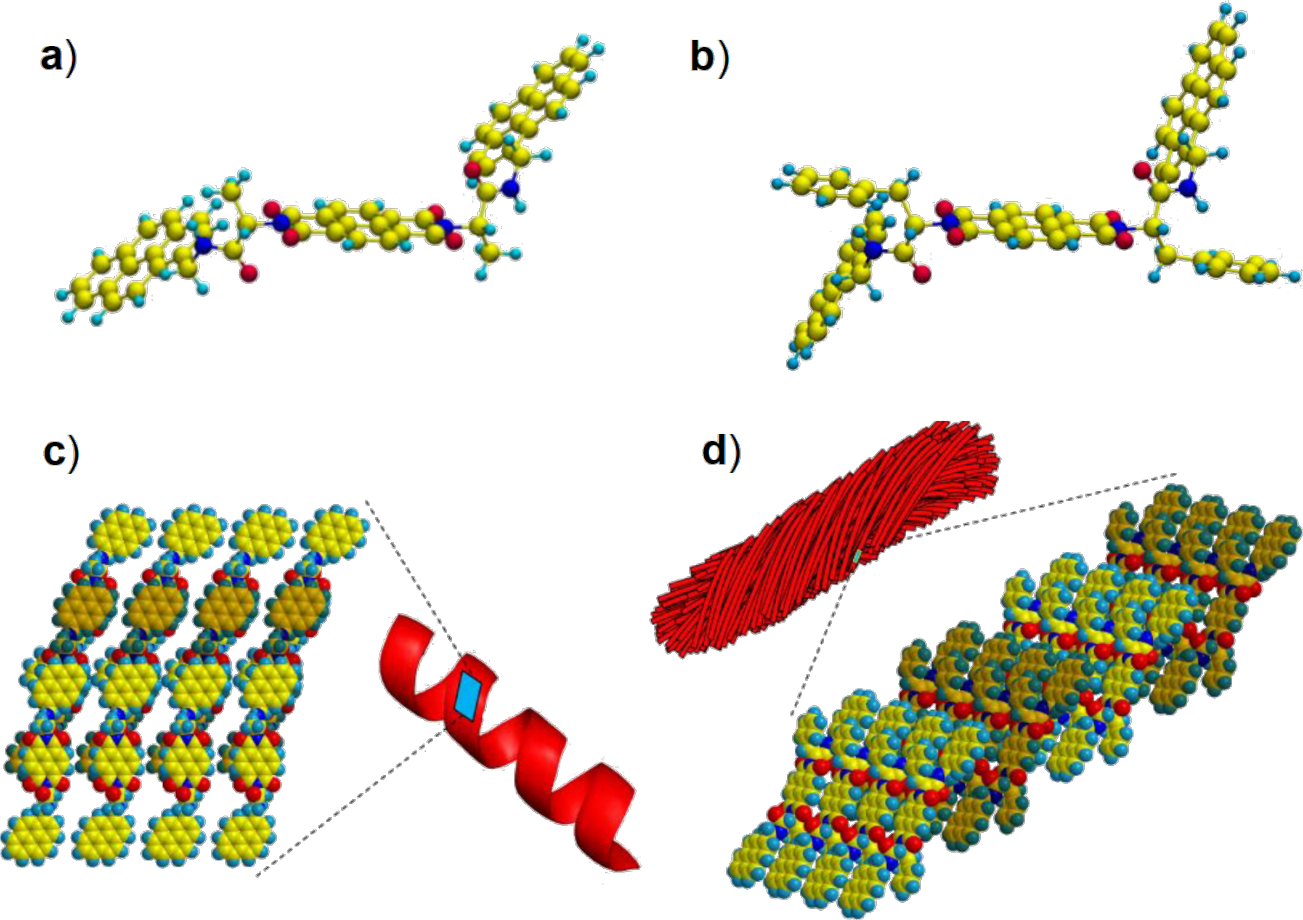
Energy-minimized structures of (a) **1** and (b) **2**. Proposed molecular stacking (Chem3D; space filling model) of topologically symmetric π-complementary pyrene and NDI of (c) **1** and (d) **2** under enhanced hydrophobic forces in aqueous solution. In order to facilitate clarity, the successive layers of D-A-D triads are shown with different contrast in (c) and (d).

In these triads, the primary molecular interaction for the assembly is charge-transfer complexation. The amino acids, which are employed to link the donor (pyrene) and acceptor (NDI) modules can also influence the assembly due to its hydrophobicity, steric bulkiness and helical propensity amongst others. So when there is intermolecular stacking of donors and acceptors, the interlinking amino acid plays the crucial role in determining the extent of charge-transfer complexation and the nature of assembly in general. In the case of alanine derivative **1**, the amino acid side chain is a methyl group (surface area: 86 Å^2^), which renders minimal steric repulsion for the intermolecular D-A stacking due to its smaller size [49]. On the other hand, the phenyl group (surface area: 194 Å^2^) in **2** renders relatively stronger steric repulsion for intermolecular D-A stacking, as evident from weaker CD signals (Figure 3c, 3d). Similarly, the side chain of isoleucine (surface area: 155 Å^2^) in **3** also facilitates weaker D-A stacking (Figure 3e, 3f). Unlike **1**, the much favoured orthogonal charge-transfer interactions of D and A are hindered in the cases of **2** and **3**, and hence would be expected to result in random architectures. However, the presence of the phenyl functionality in **2** is believed to facilitate some favourable aromatic interactions with NDI and pyrene modules, and thereby results in hierarchical helical bundles. Further, energy minimized molecular structures of the triads, **1** and **2** were obtained by using the Gaussian-09 software (Hartree–Fock method with 3-21G basis set) to get additional insights about their conformations (Figure 5a, 5b). After careful evaluation of absorption, fluorescence, circular dichroism, NMR as well as PXRD data, the plausible molecular stacking model depicting the charge-transfer complexation under enhanced hydrophobic forces thus obtained is shown in Figure 5c and 5d.

## Conclusion

In conclusion, we have designed and elucidated the molecular assembly of amino acid interlinked novel D-A-D triads. Herein, a relatively weaker noncovalent interaction like charge-transfer complexation has been achieved by employing a topologically symmetric and π-complementary pyrene and NDI. Consequently, free-floating high-aspect-ratio supercoiled nanofibres and hierarchical helical bundles of triads are realized by modulating the chemical functionality of interlinking amino acids. Such molecular-recognition processes embodied in pertinent designer functional molecules are believed to pave the way for programmable molecular assemblies with advanced applications.

## Supporting Information

File 1 Experimental details, synthesis procedures, FESEM images, absorption, photoluminescence, PXRD and 2D NOESY spectra.
