# Supplementary material for: Molecular assembly of amino acid interlinked, topologically symmetric, π-complementary donor–acceptor–donor triads
Source: Beilstein J Org Chem. 2013 Aug 1;9:1565–71. doi: 10.3762/bjoc.9.178 (PMC3740681; doi:10.3762/bjoc.9.178)
Supplement: File 1 — Experimental details, synthesis procedures, FESEM images, absorption, photoluminescence, PXRD and 2D NOESY spectra. [file Beilstein_J_Org_Chem-09-1565-s001.pdf]

Supporting Information

for

Molecular assembly of amino acid interlinked topologically symmetric π-complementary donor–acceptor–donor triads

M. B. Avinash, K. V. Sandeepa and T. Govindaraju*

Address: [1]Bioorganic Chemistry Laboratory, New Chemistry Unit, Jawaharlal Nehru Centre for Advanced Scientific Research, Jakkur, Bangalore-560064, India. Fax: +91 80 2208 2627; Tel: +91 80 2208 2969

Email: T. Govindaraju* - tgraju@jncasr.ac.in

*Corresponding author

Experimental details, synthesis procedures, FESEM images, absorption, photoluminescence, PXRD and 2D NOESY spectra.

Contents

1. **Materials and methods (S2)**
2. **Synthesis procedures (S3–S6)**
3. **Absorption and photoluminescence spectra (S6–S8)**
4. **FESEM images (S9–S11)**
5. **Powder X-ray diffraction study (S12)**
6. **2D NOESY (S13–S16)**

1. Materials and methods

Materials: 1,4,5,8-Naphthalenetetracarboxylic acid dianhydride, 1-pyrenemethylamine hydrochloride and *N*,*N*-diisopropylethylamine were obtained from Sigma-Aldrich, and 1-hydroxybenzotriazole, L-alanine, L-phenylalanine and L-isoleucine from Spectrochem Pvt. Ltd. (Mumbai, India). All other reagents and solvents were of reagent grade and used without further purification.

NMR Spectroscopy, Mass Spectrometry (MS), and Elemental Analysis: ^{1}H, ^{13}C and 2D NOESY NMR spectra were recorded on a Bruker AV-400 spectrometer with chemical shifts reported as ppm (in DMSO-d_6 with tetramethylsilane as internal standard). Mass spectra were obtained from a Shimadzu 2020 LC-MS. Elemental analysis was carried out on a ThermoScientific FLASH 2000 Organic Element Analyzer.

Absorption Spectroscopy: UV–vis spectra were recorded on a Perkin Elmer Model Lambda 900 spectrophotometer. 200 µM solutions of the samples were analyzed in quartz cuvettes of 1 mm path length.

Fluorescence Spectroscopy: Fluorescence spectra were recorded on a Perkin Elmer Model LS 55 spectrophotometer. 200 µM solutions of the samples were analyzed in a quartz cuvettes of 1 mm path length with an excitation at 345 nm.

Circular Dichroism (CD): CD measurements were carried out on a Jasco J-815 spectropolarimeter under nitrogen atmosphere. 400 µM solutions of the samples were analyzed in quartz cuvettes of 1 mm path length.

Field Emission Scanning Electron Microscopy: FESEM images were acquired with a FEI Nova nanoSEM-600 equipped with a field-emission gun operating at 15 kV. The samples were prepared by drop-casting free floating aggregates onto a Si (111) substrate.

Powder X-ray Diffraction (PXRD): PXRD patterns were recorded with a Rigaku-99 (Miniflex) diffractometer using Cu Kα radiation (λ = 1.5406 Å). The free-floating self-assembled aggregates formed at 40% aqueous DMSO or 40% aqueous NMP were drop casted on a glass slide. The diffraction peaks were indexed by DICVOL program.

Computational Details: Energy minimized structures of **1** and **2** were obtained using the software Gaussian-09. The optimization was carried out by the Hartree–Fock method and 3-21G basis set. The optimized geometries were visualized using Visual Molecular Dynamics (VMD).

2. Synthesis procedures

Synthesis of 1

*Synthesis of alanine appended NDI (*Ala-NDI-Ala*)*: 1,4,5,8-naphthalenetetracarboxylic acid dianhydride (100 mg, 0.373 mmol) and L-alanine (66.46 mg, 0.746 mmol) taken in 20 mL of *N*,*N*-dimethylformamide were sonicated for 5 min. The resulting suspension was heated for 13 h at 110 °C and then the solvent was concentrated to 5 mL under reduced pressure. The residue was purified by precipitation and washing with diethyl ether to afford a yellow solid in quantitative yield.

Compound Ala-NDI-Ala (100 mg, 0.244 mmol), 1-ethyl-3-(3-dimethylaminopropyl)carbodiimide (102.8 mg, 0.54 mmol) and hydroxybenzotriazole (72.96 mg, 0.54 mmol) were dissolved in *N*,*N*-dimethylformamide (10 mL) and stirred in an ice bath for about 15 min under inert atmosphere. 1-Pyrenemethylamine hydrochloride (131 mg, 0.5 mmol) and diisopropylethyl amine (0.3 mL) were added to the reaction mixture. The resulting solution was stirred at room temperature for 24 h and the red

coloured precipitate was filtered and washed with excess methanol to afford the red solid, **1** in 63% yield. ^{1}H NMR (400 MHz, DMSO-d_6, δ in ppm): 8.63(m, 6H), 8.12(m, 18H), 5.59(q, 2H), 4.99(t, 4H), 1.63(d, 6H). ^{13}C NMR (100 MHz. DMSO-d_6, δ in ppm): 168.91, 162.45, 132.58, 130.69, 130.22, 129.93, 127.96, 127.42, 127.30, 126.88, 126.58, 126.47, 126.19, 125.12, 125.07, 124.49, 123.84, 123.78, 123.11, 49.90, 41.00, 14.35. EIMS: *m*/*z* = 836.31 $[M]^+$ for $C_{54}H_{36}N_4O_6$. Elemental analysis: Found: C, 77.47; H, 4.40; N, 6.62; calcd: C, 77.50; H, 4.34; N, 6.69 for $C_{54}H_{36}N_4O_6$.

Synthesis of 2

*Synthesis of phenylalanine appended NDI (*Phe-NDI-Phe*)*: 1,4,5,8-naphthalenetetracarboxylic acid dianhydride (100 mg, 0.373 mmol) and L-phenylalanine (124 mg, 0.746 mmol) taken in 20 mL of *N*,*N*-dimethylformamide was sonicated for 5 min. The resulting suspension was heated for 16 h at 110 °C and then the solvent was concentrated to 5 mL under reduced pressure. The residue was purified by precipitation and washing with diethyl ether to afford a brown solid in quantitative yield.

Compound Phe-NDI-Phe (70 mg, 0.124 mmol), 1-ethyl-3-(3-dimethylaminopropyl)carbodiimide (52.52 mg, 0.274 mmol), hydroxybenzotriazole (37 mg, 0.274 mmol) was dissolved in *N*,*N*-dimethylformamide (10 mL) and stirred in an ice bath for about 15 min under inert atmosphere. 1-Pyrenemethylamine hydrochloride (67 mg, 0.248 mmol) and diisopropylethyl amine (0.5 mL) were added to the reaction mixture. The resulting solution was stirred at room temperature for 24 h and the red coloured precipitate was filtered and washed with excess methanol to afford the red solid, **2** in 52% yield. ^{1}H NMR (400 MHz, DMSO-d_6, δ in ppm): 8.72(t, 2H), 8.53(s, 4H),

8.13(m, 18H), 5.86(q, 2H), 5.02(m, 4H), 3.73(dd, 2H), 3.38(dd, 2H). ^{13}C NMR (100 MHz. DMSO-d_6, δ in ppm): 168.12, 162.39, 137.97, 132.49, 130.72, 130.37, 130.22, 129.96, 128.91, 127.97, 127.92, 127.45, 127.32, 126.91, 126.17, 125.84, 125.15, 125.07, 124.53, 123.88, 123.80, 123.06, 55.43, 40.91, 33.83. EIMS: *m*/*z* = 988.48 $[M]^+$ for $C_{66}H_{44}N_4O_6$. Elemental analysis: Found: C, 80.21; H, 4.54; N, 5.61; calcd: C, 80.15; H, 4.48; N, 5.66 for $C_{66}H_{44}N_4O_6$.

Synthesis of 3

Synthesis of isoleucine appended NDI (Ile-NDI-Ile): 1,4,5,8-naphthalenetetracarboxylic acid dianhydride (100 mg, 0.373 mmol) and L-isoleucine (98 mg, 0.746 mmol) taken in 20 mL of *N*,*N*-dimethylformamide were sonicated for 5 min. The resulting suspension was heated for 24 h at 110 °C and then the solvent was concentrated to 5 mL under reduced pressure. The residue was purified by precipitation and washing with diethyl ether to afford a brown solid in quantitative yield.

Compound Ile-NDI-Ile (400 mg, 0.809 mmol), 1-ethyl-3-(3-dimethylaminopropyl)carbodiimide (342 mg, 1.781 mmol) and hydroxybenzotriazole (241 mg, 1.781 mmol) were dissolved in *N*,*N*-dimethylformamide (15 mL) and stirred in an ice bath for about 15 min under an inert atmosphere. 1-Pyrenemethylamine hydrochloride (437 mg, 1.618 mmol) and diisopropylethyl amine (0.5 mL) were added to the reaction mixture. The resulting solution was stirred at room temperature for 24 h and the red coloured precipitate was filtered and washed with an excess of methanol to afford the red solid, **3** in 55% yield. ^{1}H NMR(400 MHz, DMSO-d_6, δ in ppm): 8.62(m, 6H), 8.13(m, 18H), 5.18(d, 2H), 4.96(m, 4H), 2.60(m, 2H), 1.19(m,

8H), 0.85(m, 2H), 0.73(t, 6H). ^{13}C NMR(100 MHz. DMSO-d_6, δ in ppm): 167.96, 162.74, 132.63, 130.73, 130.66, 130.17, 129.90, 127.97, 127.32, 126.84, 126.56, 126.19, 126.12, 125.07, 124.99, 124.40, 123.84, 123.76, 123.07, 58.65, 40.87, 32.66, 24.46, 18.59, 11.09. EIMS: m/z = 920.45 $[M]^+$ for $C_{60}H_{48}N_4O_6$. Elemental analysis: Found: C, 78.31; H, 5.34; N, 6.11; calcd: C, 78.24; H, 5.25; N, 6.08 for $C_{60}H_{48}N_4O_6$.

3. Absorption and photoluminescence spectra

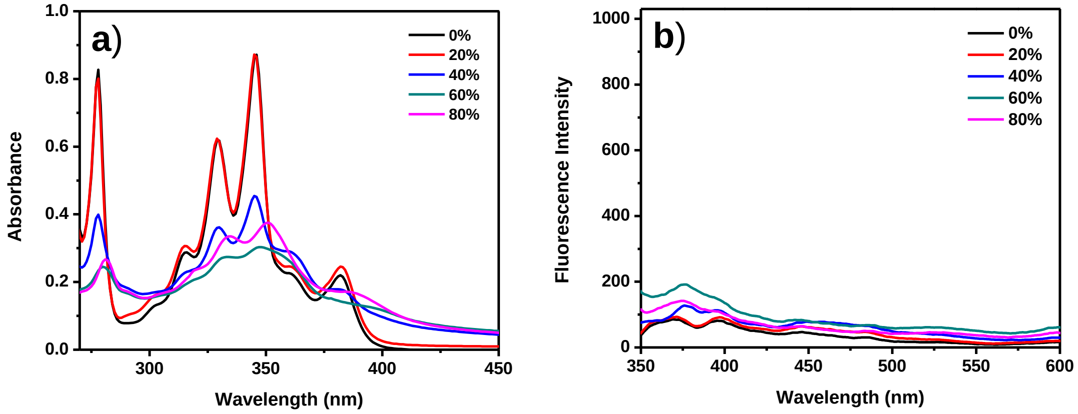

Figure S1: (a) UV–vis and (b) fluorescence spectra of 200 μM of **1** in aqueous NMP. Excitation at 345 nm. The values represent the various percentages of water in aqueous NMP.

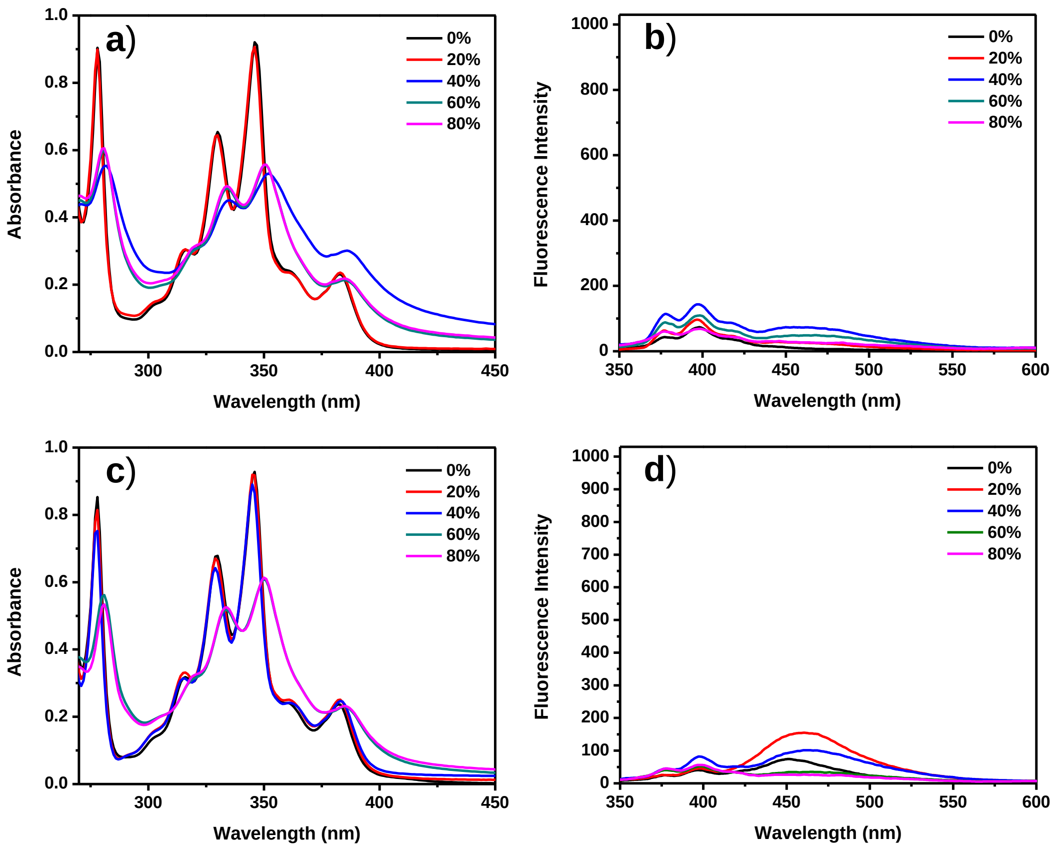

Figure S2: (a) and (c) UV–vis, (b) and (d) fluorescence spectra of 200 μM of **2**. (a) and (b) are in aqueous DMSO, (c) and (d) are in aqueous NMP. Excitation at 345 nm. The values represent the various percentages of water in aqueous NMP.

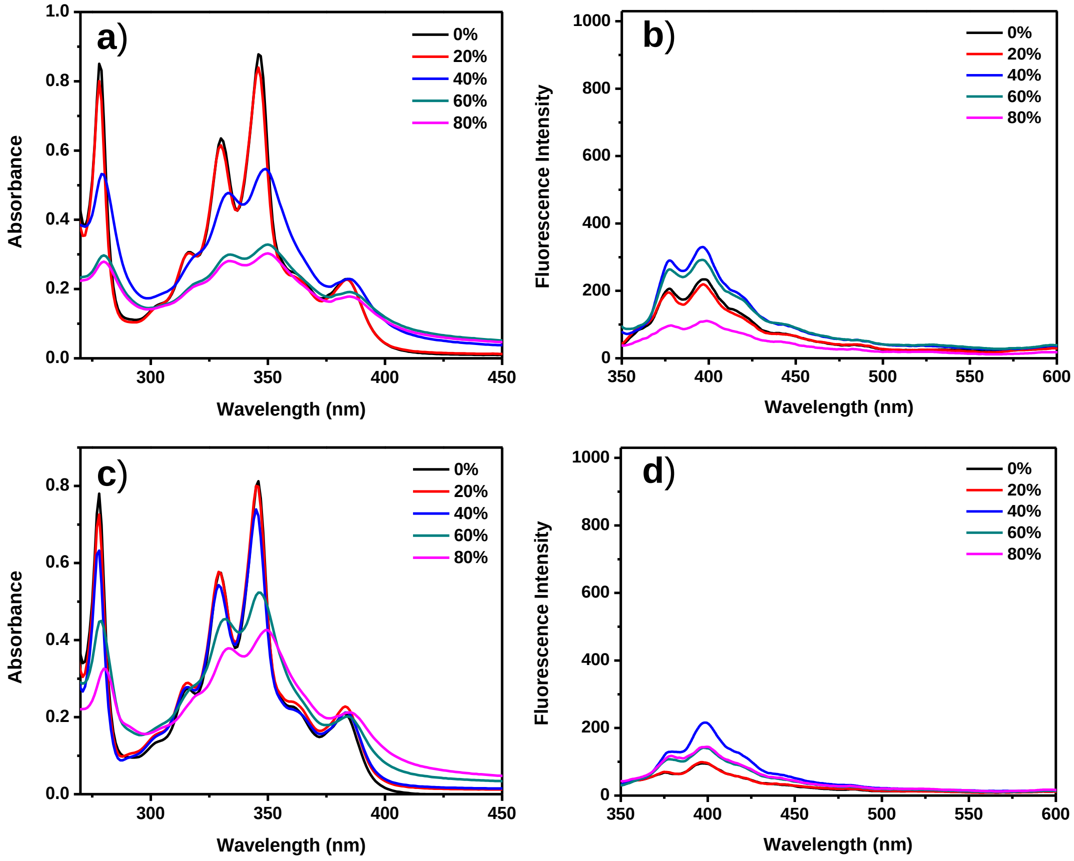

Figure S3: (a) and (c) UV–vis, (b) and (d) fluorescence spectra of 200 μM of **3**. (a) and (b) are in aqueous DMSO, (c) and (d) are in aqueous NMP. Excitation at 345 nm. The values represent the various percentages of water in aqueous NMP.

4. FESEM images

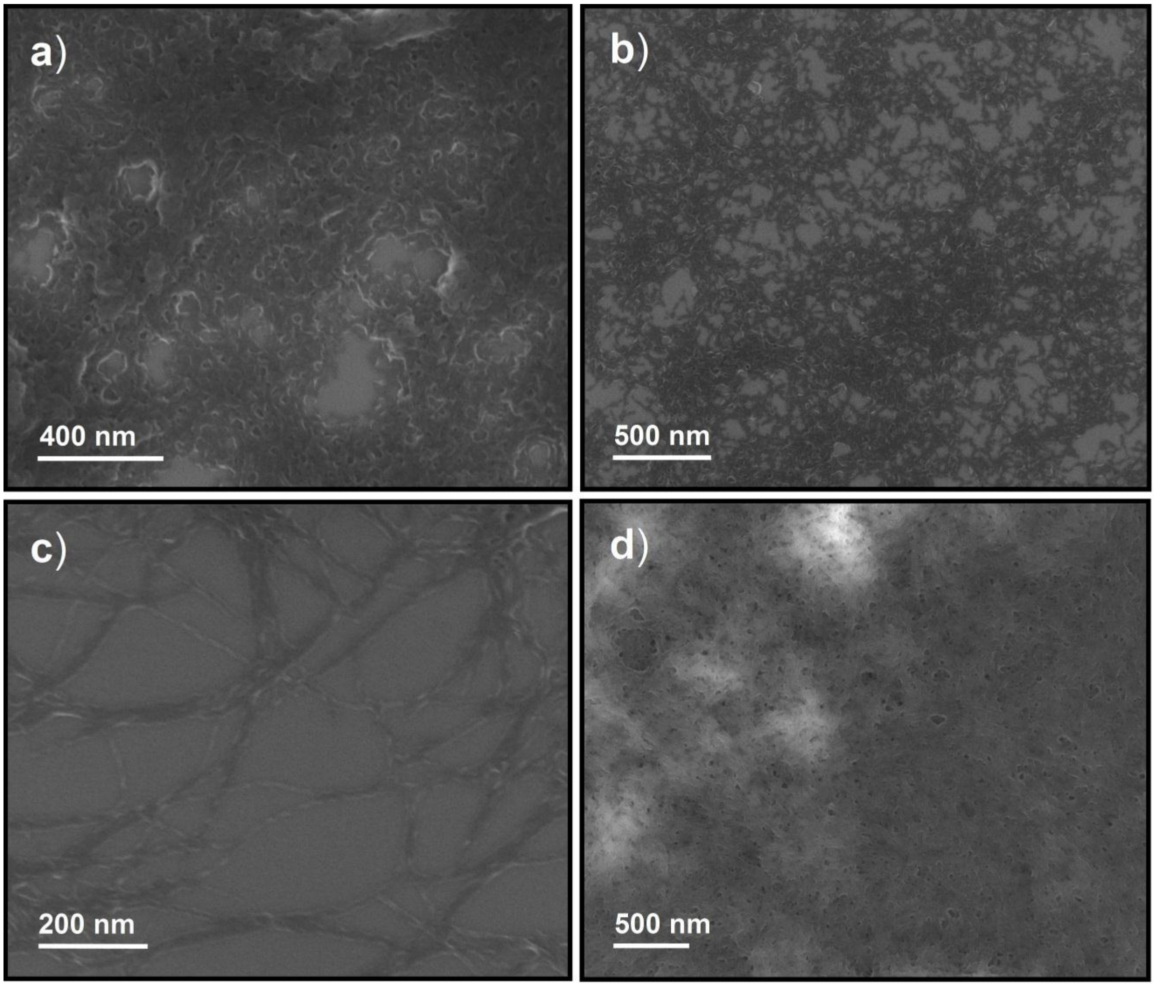

Figure S4: FESEM image of **1** obtained from (a) 40% and (b) 80% aqueous DMSO. FESEM image of **1** obtained from (c) 40% and (d) 80% aqueous NMP.

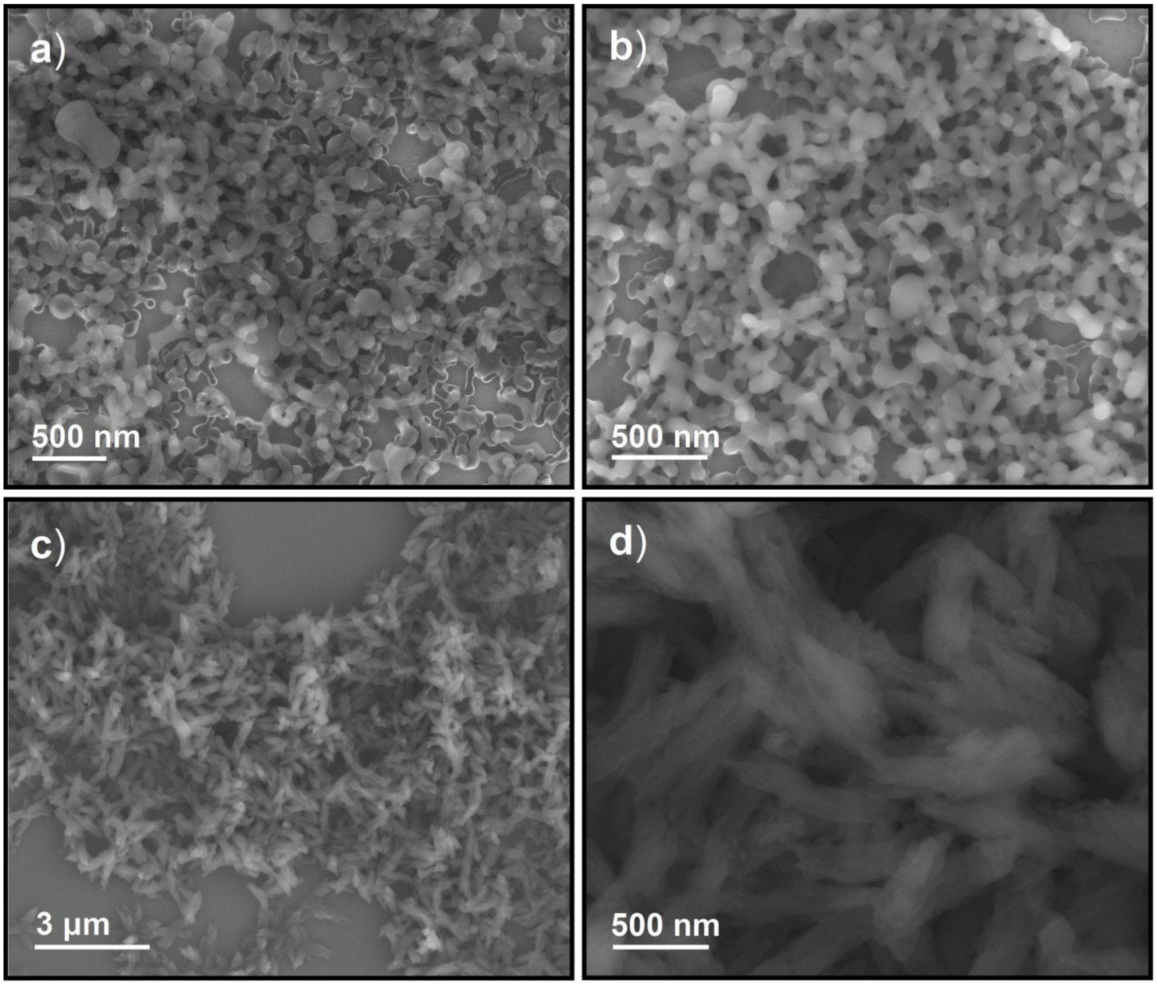

Figure S5: FESEM image of **2** obtained from (a) 40% and (b) 80% aqueous DMSO. (c) and (d) FESEM image of **2** obtained from 80% aqueous NMP.

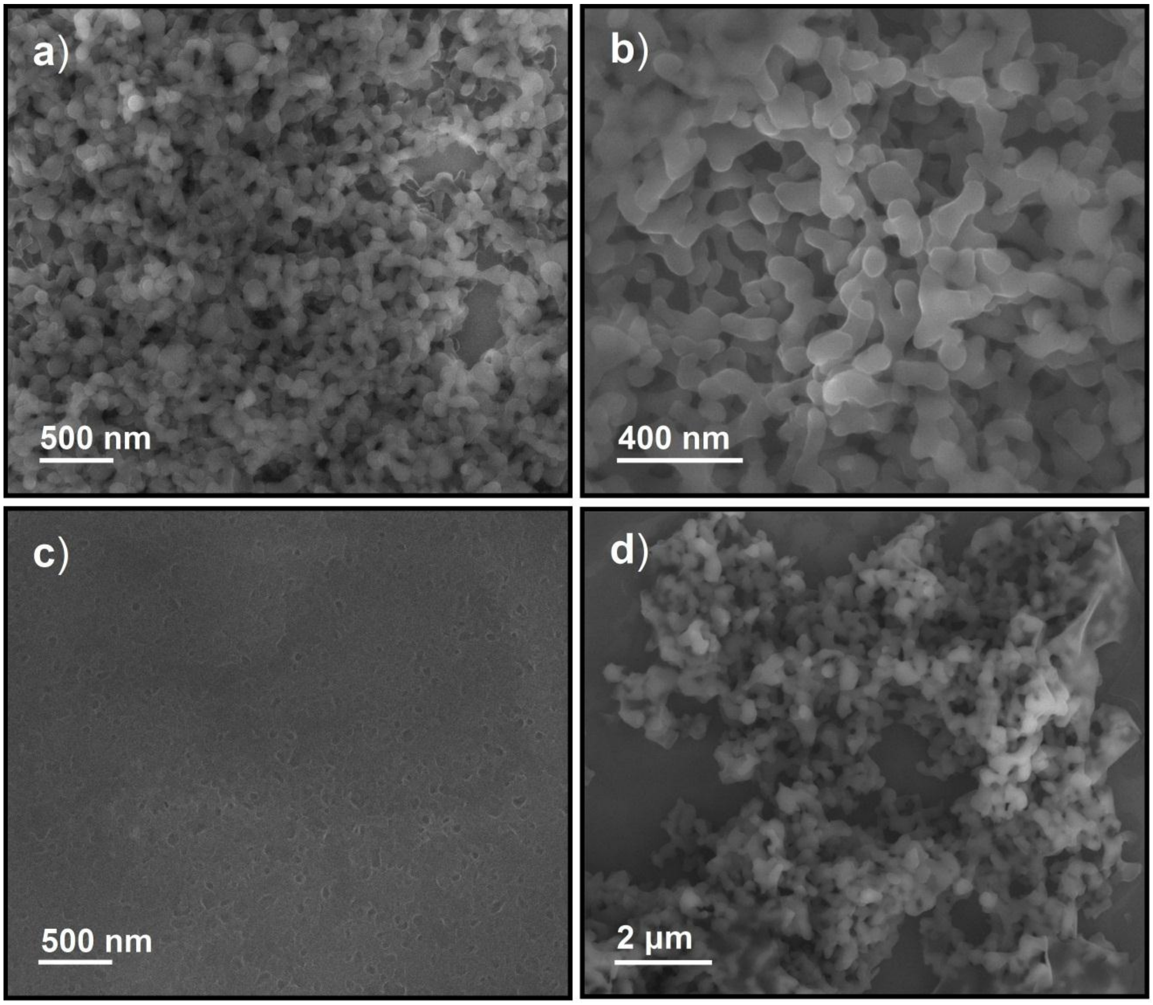

Figure S6: FESEM image of **3** obtained from (a) 40% and (b) 80% aqueous DMSO. FESEM image of **3** obtained from (c) 40% and (d) 80% aqueous NMP.

5. Powder X-ray diffraction study

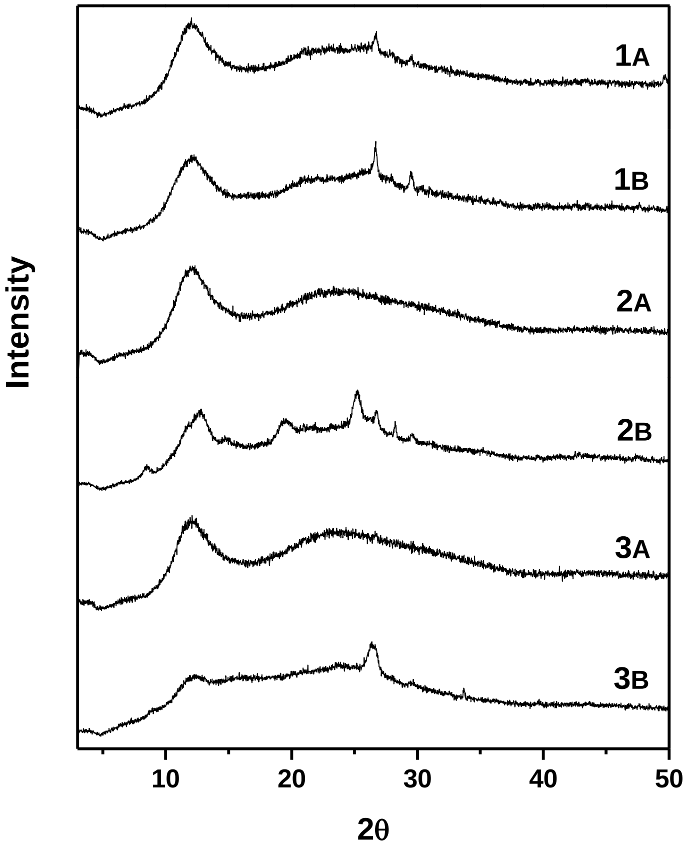

Figure S7: PXRD of **1**, **2** and **3** obtained from 40% aqueous DMSO (A) and 40% aqueous NMP (B) respectively.

6. 2D NOESY

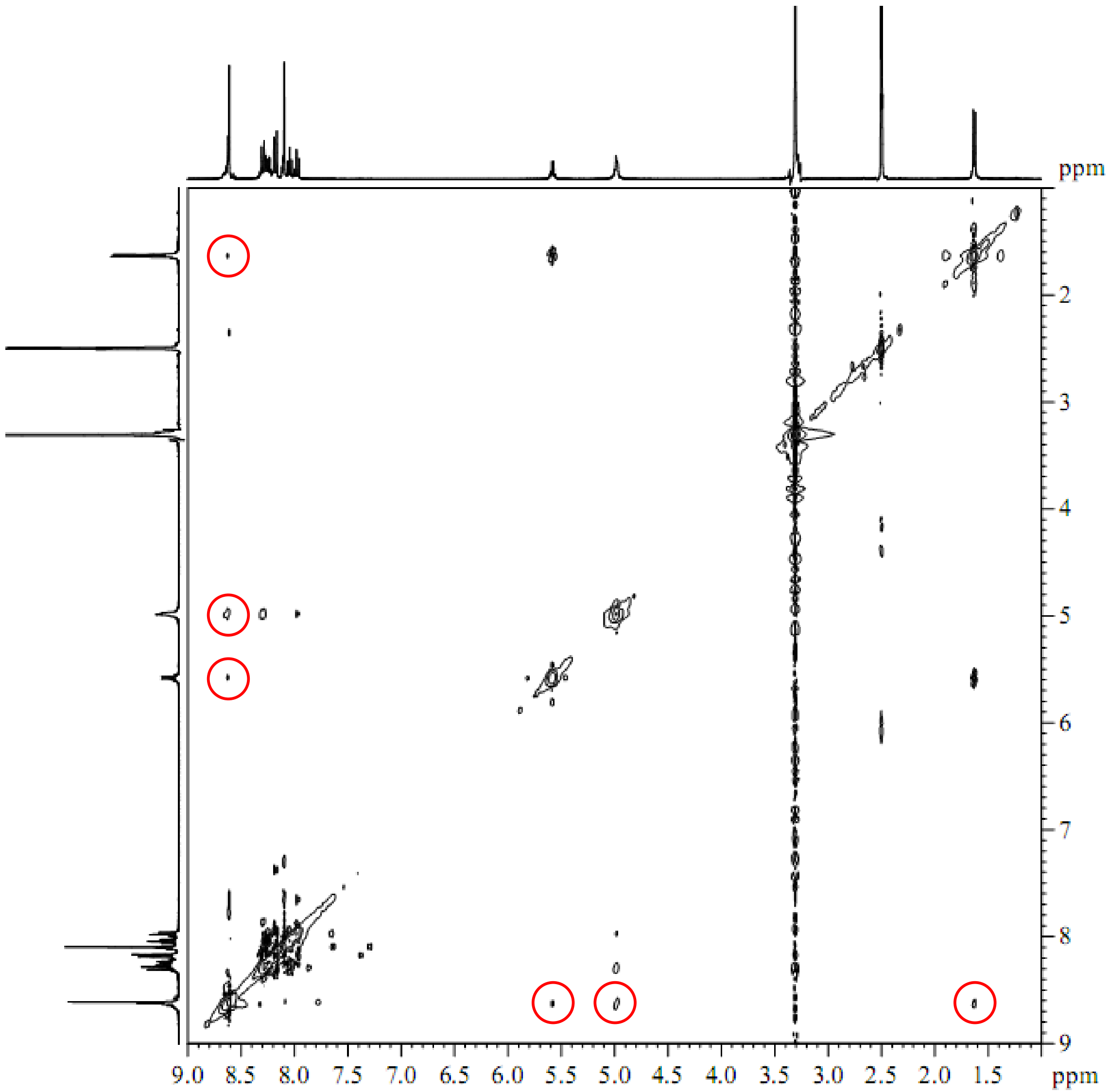

Figure S8: 2D NOESY NMR spectra of **1**. Red circles indicate the cross peaks of amide NH proton with the proton of α-carbon and CH_3 protons of alanine as well as with the CH_2 protons of pyrenemethylamine.

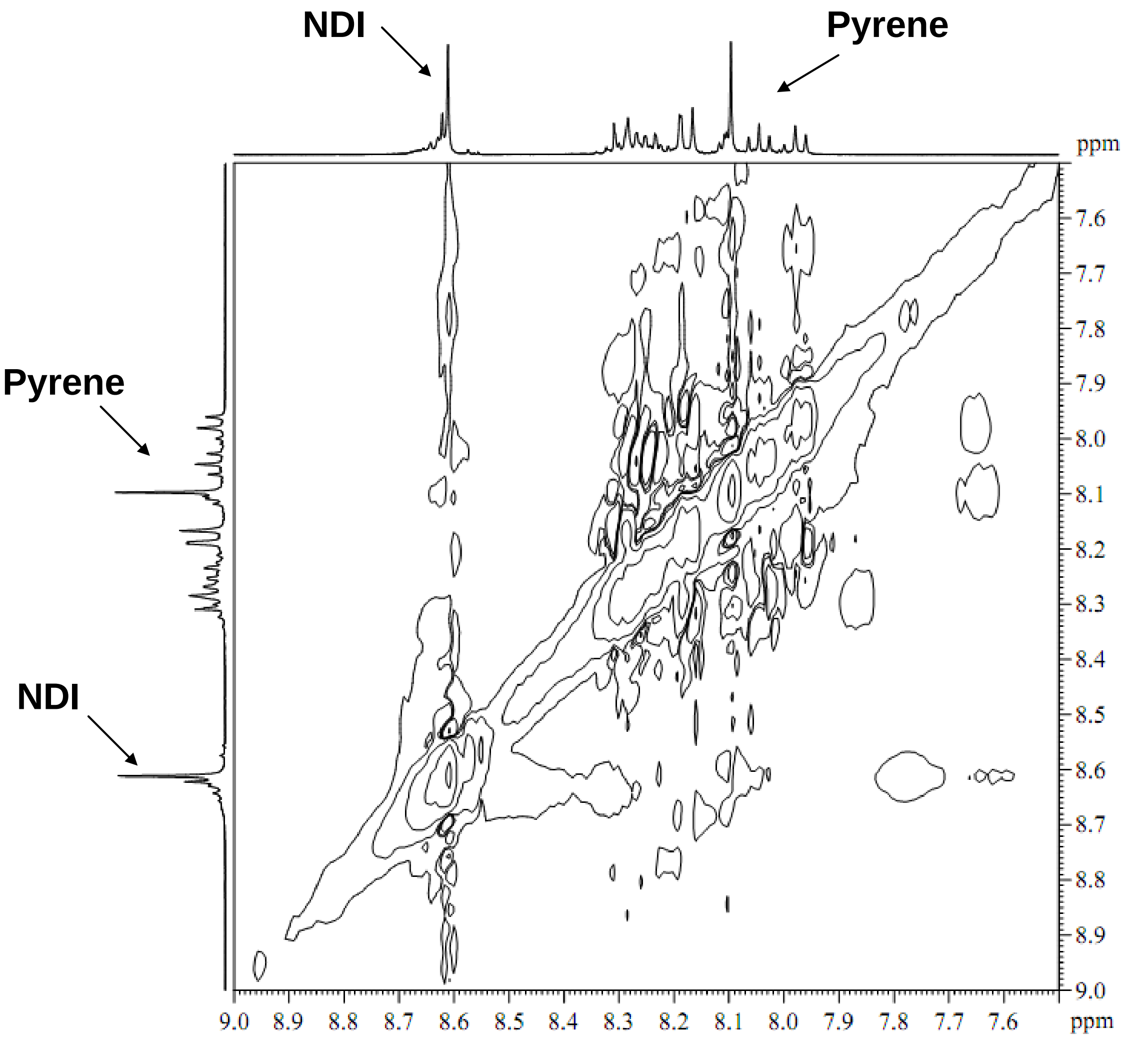

Figure S9: 2D NOESY NMR spectra of **1** shows a weak spatial aromatic interaction of NDI and pyrene in DMSO-d_6.

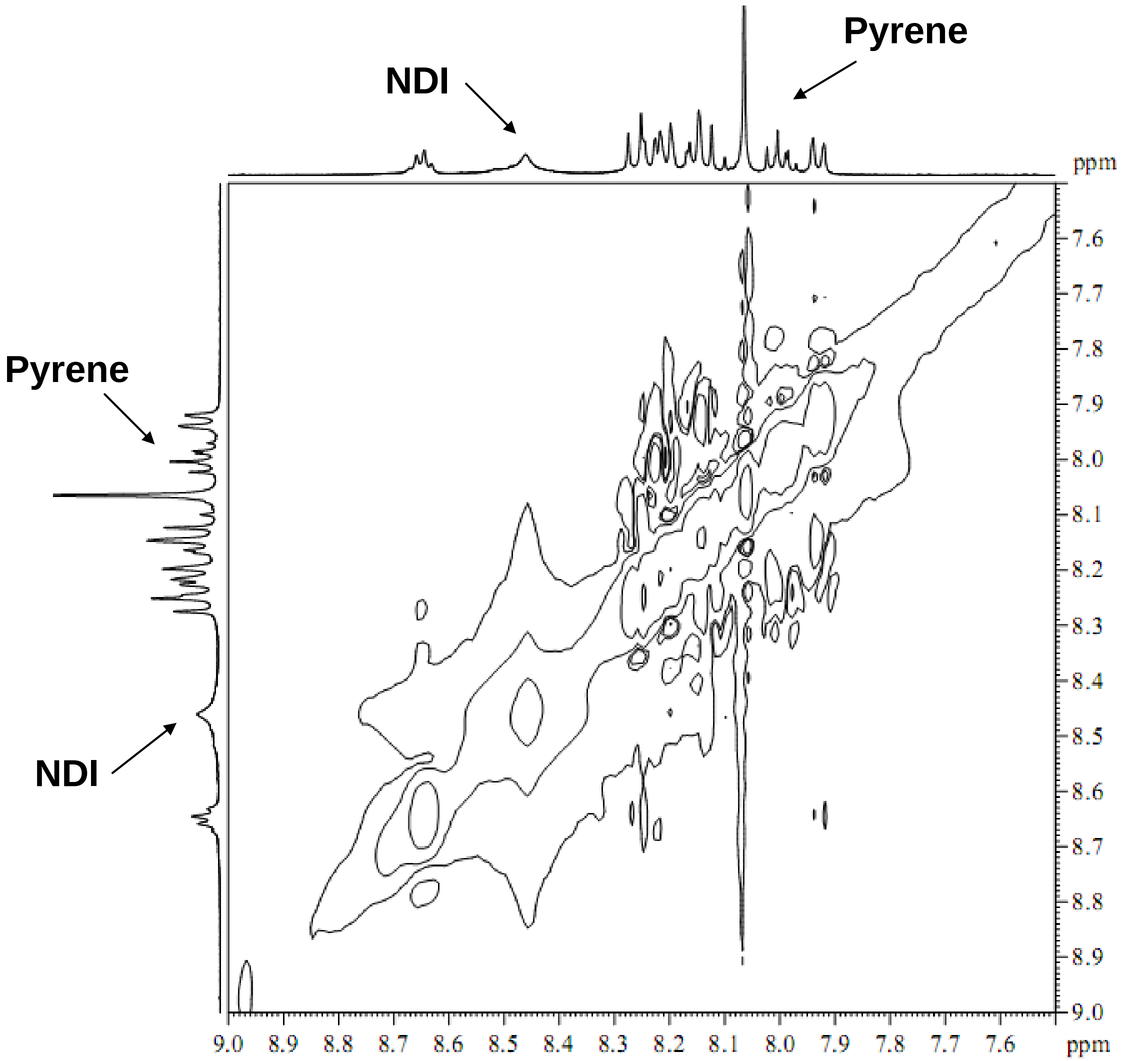

Figure S10: 2D NOESY NMR spectra of **2** shows a weak spatial aromatic interaction of NDI and pyrene in DMSO-d_6.

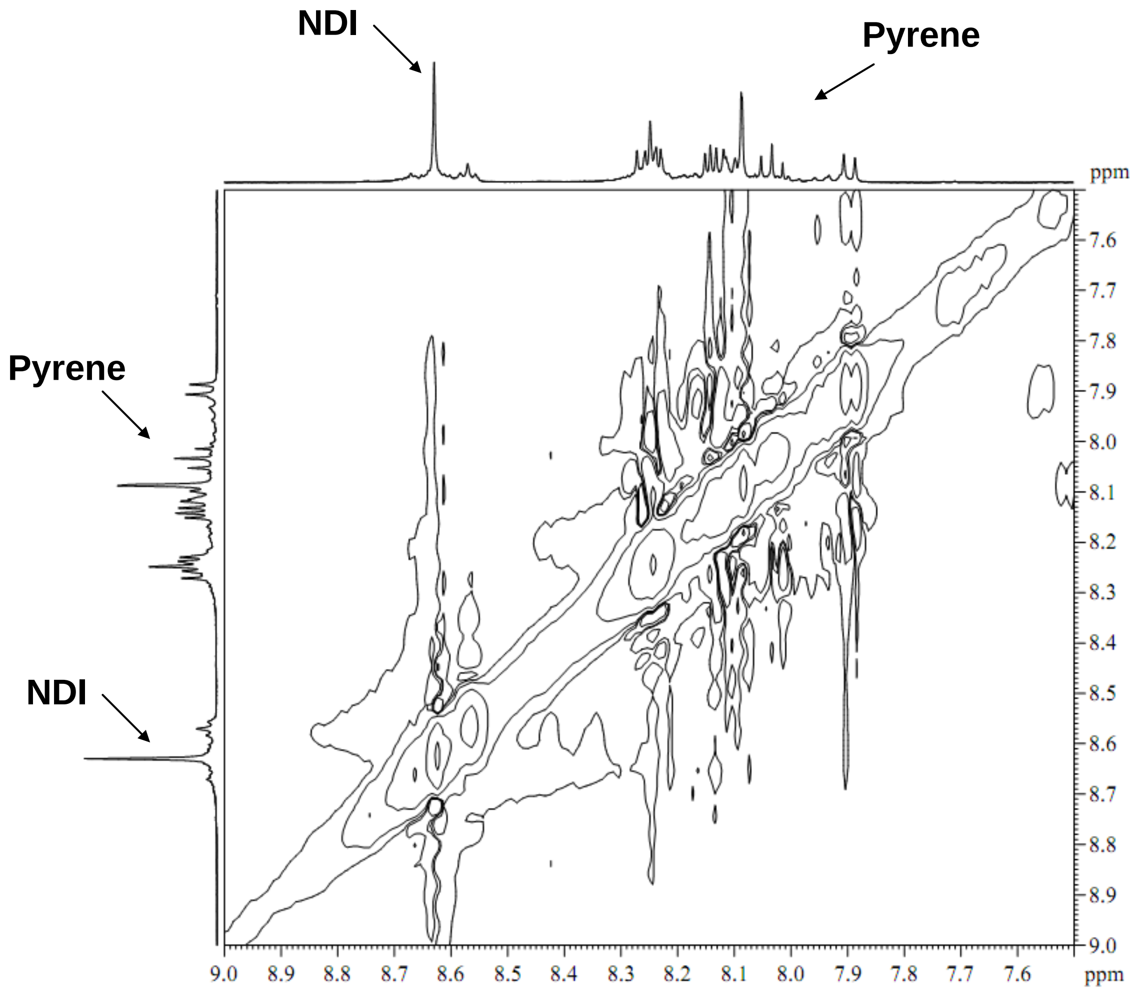

Figure S11: 2D NOESY NMR spectra of **3** shows a weak spatial aromatic interaction of NDI and pyrene in DMSO-d_6.
